# Genetic Mapping of *ms1s*, a Recessive Gene for Male Sterility in Common Wheat

**DOI:** 10.3390/ijms22168541

**Published:** 2021-08-09

**Authors:** Wenlong Yang, Yafei Li, Linhe Sun, Muhammad Shoaib, Jiazhu Sun, Dongzhi Wang, Xin Li, Dongcheng Liu, Kehui Zhan, Aimin Zhang

**Affiliations:** 1 State Key Laboratory of Plant Cell and Chromosome Engineering, Institute of Genetics and Developmental Biology/Innovative Academy of Seed Design, Chinese Academy of Sciences, Beijing 100101, China; yafeili2019@126.com (Y.L.); shoaib.cas@hotmail.com (M.S.); jzsun@genetics.ac.cn (J.S.); wangdongzhi1990@163.com (D.W.); lixin@genetics.ac.cn (X.L.); dcliu@genetics.ac.cn (D.L.); 2 Institute of Vegetables and Flowers, Chinese Academy of Agricultural Sciences, Beijing 100081, China; 3 College of Agronomy, Henan Agricultural University, Zhengzhou 450002, China; kh486@163.com; 4 Institute of Botany, Jiangsu Province and Chinese Academy of Sciences (Nanjing Botanical Garden Mem. Sun Yat-Sen), Nanjing 210014, China; linhesun@cnbg.net; 5 University of Chinese Academy of Sciences, Beijing 100049, China

**Keywords:** genic male sterility, genetic mapping, CAPS marker, *Triticum aestivum*

## Abstract

The utilization of heterosis is an important way to improve wheat yield, and the production of wheat hybrid seeds mainly relies on male-sterile lines. Male sterility in line 15 Fan 03 derived from a cross of 72,180 and Xiaoyan 6 is controlled by a single recessive gene. The gene was mapped to the distal region of chromosome 4BS in a genetic interval of 1.4 cM and physical distance of 6.57 Mb between SSR markers Ms4BS42 and Ms4BS199 using an F_2_ population with 1205 individuals. Sterile individuals had a deletion of 4.57 Mb in the region presumed to carry the *Ms1* locus. The allele for sterility was therefore named *ms1s.* Three CAPS markers were developed and verified from the region upstream of the deleted fragment and can be used for *ms1s* marker-assisted selection in wheat hybrid breeding. This work will enrich the utilization of male sterility genetic resources.

## 1. Introduction

As a major food crop, common wheat (*Triticum aestivum* L., AABBDD) plays a vital role in ensuring the food security of human beings. Development of wheat semi-dwarf varieties during the 1950s and 1960s improved lodging resistance and permitted significant increases in yield that led to a stable global food supply and eliminated the threat of hunger. These events became known as the “Green Revolution”. However, with arable land currently decreasing due to urbanization, environmental degradation, and rising population levels, there is an emerging need to further increase wheat yield. Heterosis, a common biological phenomenon widely used in maize (*Zea mays* L.) and rice (*Oryza Sativa* L.), offers a way to increase wheat yield [1,2]. Despite decades of research, the use of male sterility for the production of hybrid wheat has achieved only limited success. Wilson and Ross (1962) introduced the nucleus of common wheat into the cytoplasm of *Triticum timopheevii* to develop a common wheat T-type male-sterile line, and fertility restorer lines (T-type restorer lines) were obtained by breeding and selection [3]. Thus, a three-parent hybrid system was devised. Subsequently, new types of sterility systems, e.g., K- and V-type cytoplasmic male sterility systems, a photo-thermal sterility system, and chemical male sterilizing agents were described. However, critical biological and economic problems hindered each system, and hybrid wheat as a commercial enterprise never exceeded 0.2% of the total production [4,5].

In 1931, Bovicini first discovered the phenomenon of nuclear male sterile in wheat, and, to date, five nuclear male sterility loci have been identified, i.e., *Ms1*, *Ms2*, *Ms3*, *Ms4*, and *Ms5* [6,7,8,9,10]. Sterility alleles *ms1* and *ms5* are recessive and located on chromosome 4BS and 3AL, respectively [11,12], whereas *Ms2*, *Ms3*, and *Ms4* are dominant. *Ms2* and *Ms4* are located on chromosomes 4DS, whereas *Ms3* is on chromosome 5AS [7,8,10,13,14]. Many sterile alleles of the *Ms1* gene have been described. These presumably arose as natural mutants, such as Pugsley’s male sterile (*ms1a*, [6,15]) and LZ (*ms1g*, [16]); as X-ray-induced mutants, such as Probus male sterile (*ms1b*, [17]) and Cornerstone (*ms1c*, [18]); or as EMS induced mutants, including FS2 (*ms1d*), FS3 (*ms1e*), and FS24 (*ms1f*) [10,11]. Recently, 12 isomorphic variants of *Ms1* gene, i.e., *ms1d.1*, *ms1d.2*, *ms1h*, *ms1i*, *ms1j*, *ms1k*, *ms1l*, *ms1m*, *ms1n*, *ms1o, ms1p,* and *ms1q*, from the spring wheat variety Ningchun 4 were identified following EMS treatment [19]. Many scientists believe that nuclear male-sterile lines are ideal lines for the production of hybrid varieties, but a lack of stable male-sterile maintainers prohibits their widespread application. Although a “two-line system” [20] and an “XYZ hybrid” system using the nuclear male-sterile mutant “Cornerstone” were proposed, neither proved successful [21,22]. Huang et al. (1988, 1991) discovered a nuclear male-sterile mutant in the offspring of a cross of wheat varieties 72,180 and Xiaoyan 6 and created a blue-aleurone nuclear male-sterile maintainer system (BM system) by introducing a 4E chromosome from *Agropyron elongatum* containing a dominant blue endosperm gene and a fertility restorer gene [23,24]. The seeds of the wheat 4E monomer alien addition line are light blue. This line can produce seeds normally by selfing, and the offspring can separate about 64% non-blue seeds (nuclear male sterile), 16% light blue seeds, 16% medium blue seeds, and 4% deep blue seeds (fertile). All plants grown from non-blue seeds were male sterile, whose fertility could be restored well in F_1_ by almost any variety of bread wheat. The light blue seed is used as a maintainer for male sterility. Zhou et al. (2006) also introduced the same 4E chromosome into Lanzhou Mutant 257A (*ms1g*) and established a recessive nuclear male-sterile 4E-ms system [25]. This solved the difficulties in obtaining stable maintainer lines and producing homozygous male-sterile seeds in large quantities. It was a significant step forward in the applied research of wheat nuclear male sterility.

In this study, we mapped a nuclear male-sterile gene in line 15 Fan 03 (derived from the blue-aleurone nuclear male-sterile maintainer system) and found a deletion of chromosome fragment within the *Ms1* locus. We developed molecular markers for MAS to speed up hybrid breeding if a suitable system were to be developed.

## 2. Results

### 2.1. Sterile Plants in Line 15 Fan 03 Cannot Form Mature Pollen

**Figure 1:**
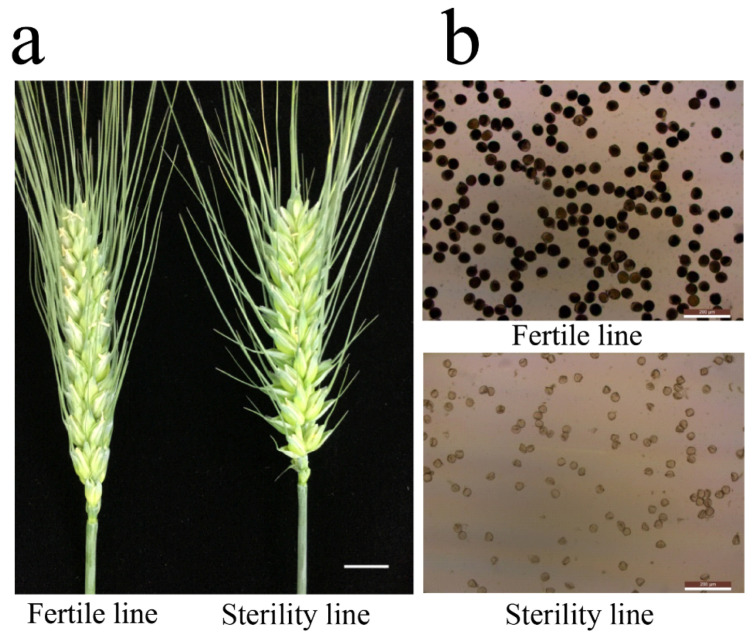
Morphological characteristics of the sterile line and fertile line. (**a**) Spikes of Mian 07-374 and a sterile plant from 15 Fan 03. Bar, 1 cm; (**b**) Pollen grains from Mian 07-374 (upper) and a sterile plant from 15 Fan 03 (lower) stained with I_2_-KI. Bar, 200 μm.

**Figure 2:**
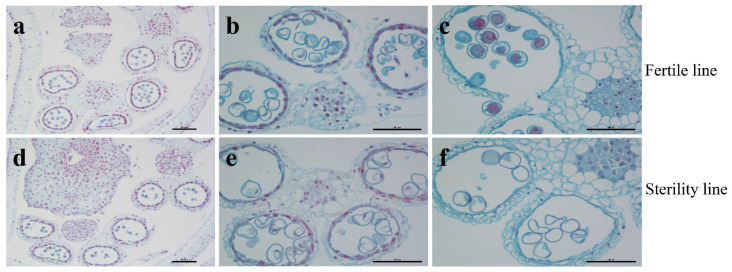
Paraffin sections of the anthers at different developmental stages in Mian 07-374 (**a**–**c**) and a sterile plant from 15 Fan 03 (**d**–**f**). Early microspore stage (**a**,**d**); late mononuclear microspore stage (**b**,**e**); mature trinuclear stage (**c**,**f**) of pollen development. Bars, 100 μm.

**Figure 3:**
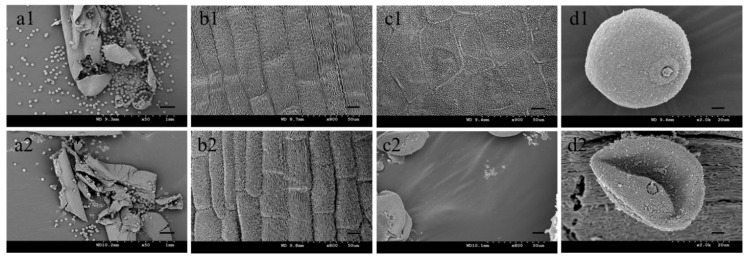
SEM observation of pollen and anthers of Mian 07-374 (**a1**–**d1**) and a sterile plant from 15 Fan 03 (**a2**–**d2**). Pollen from burst anthers. Bars, 0.2 mm (**a1**,**a2**). Anther walls. Bars, 10 μm (**b1**,**b2**). Cuticle surfaces. Bars, 10 μm (**c1**,**c2**). Pollen. Bars, 4 μm (**d1**,**d2**).

Compared to the fertile line Mian 07-374, the glumes of sterile plants in 15 Fan 03 had a larger opening angle opened for a longer time, were translucent, and had shorter filaments causing the anthers to remain in the glume. Staining with 1% I_2_-KI showed that pollen grains from sterile plants were yellowish-brown and malformed, demonstrating abnormal development and lack of fertility (Figure 1). The anthers of sterile and fertile plants were observed anatomically at different stages, and no significant differences were observed in tapetum cell structure and microspore development during the mononucleosis stages; however, degradation became evident at the tri-nuclear stage (Figure 2). Furthermore, at the tri-nuclear stage, the sterile line had less pollen grains compared to the fertile line; this was observed by scan electron microscopy (SEM) (Figure 3). There were no discernible differences in the surface pattern on the anther walls between the two lines; however, the inner anther walls of pollen grains in sterile plants were smooth and lacked grainy ubisch bodies, whereas granulated ubisch bodies separated by similar cellular structures were densely distributed on the inner walls of pollen from the fertile line. Pollen grains from the fertile line were plump and near-spherical with a bulged ring protruding from the germination hole. By contrast, the aborted pollen grains from sterile plants were collapsed, irregular, and deformed; the germination hole was caved-in. This implies that sterile pollens are not fully developed and cannot pollinate normally.

### 2.2. The Nuclear Male Sterility Gene Is Located on Chromosome 4BS

**Figure 4:**
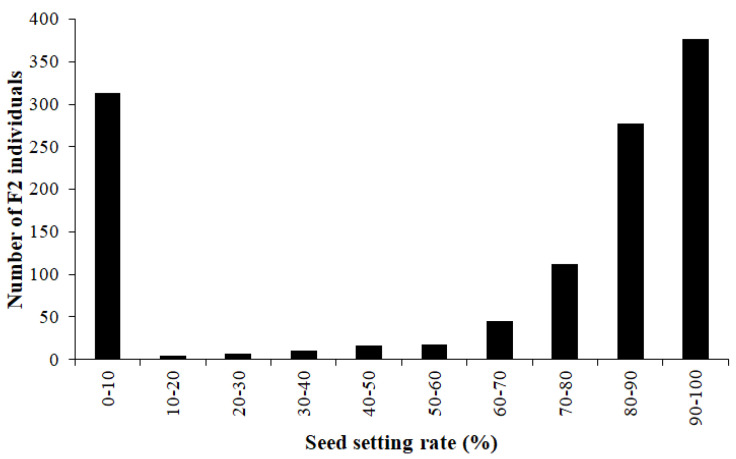
The seed setting rates of the F_2_ population.

The F_1_ generation of the cross between a male-sterile plant in 15 Fan 03 and Mian 07-374 showed 100% fertility. The F_2_ population derived from F_1_ plants segregated 892 fertile: 313 sterile, conforming with a 3:1 Mendelian ratio (*χ*^2^ = 0.458, *p* = 0.498) and indicating that sterility was conferred by a recessive gene (Figure 4).

**Figure 5:**
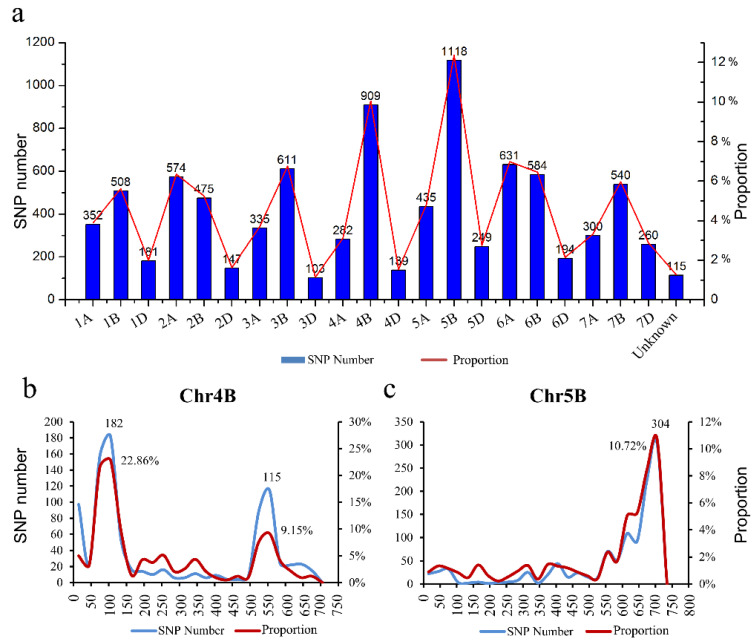
SNP distribution across all wheat chromosomes (**a**) and chromosomes 4B (**b**) and 5B (**c**).

DNA from the pooled samples of 15 Fan 03 (male-sterile line), Mian 07-374 (fertile line), F_2_ individuals with 0% seed set (homozygous recessive, msms), and F_2_ individuals with more than 99% seed set (heterozygous and homozygous dominant, Msms and MsMs) was used as a template for whole-genome SNP genotyping using the Wheat660 SNP chip. Of a total of 9042 SNPs detected homozygous in both 15 Fan 03 and 0% seed set pool, 10.05% (909) were located on chromosome 4B, whereas 1118 (12.36%) were located on chromosome 5B. Less than 7% were located on each of the remaining chromosomes, and the locations of 115 (1.27%) were not predictable (Figure 5a). For chromosome 4B, 22.86% of the SNPs were located in the 0–150 Mb region, and 9.15% were in the 500–600 Mb region; for chromosome 5B 10.72% of the SNPs were located in the 640–730 Mb region (Figure 5b,c). These results predicted that the gene responsible for male sterility could be on chromosome 4B or 5B.

**Figure 6:**
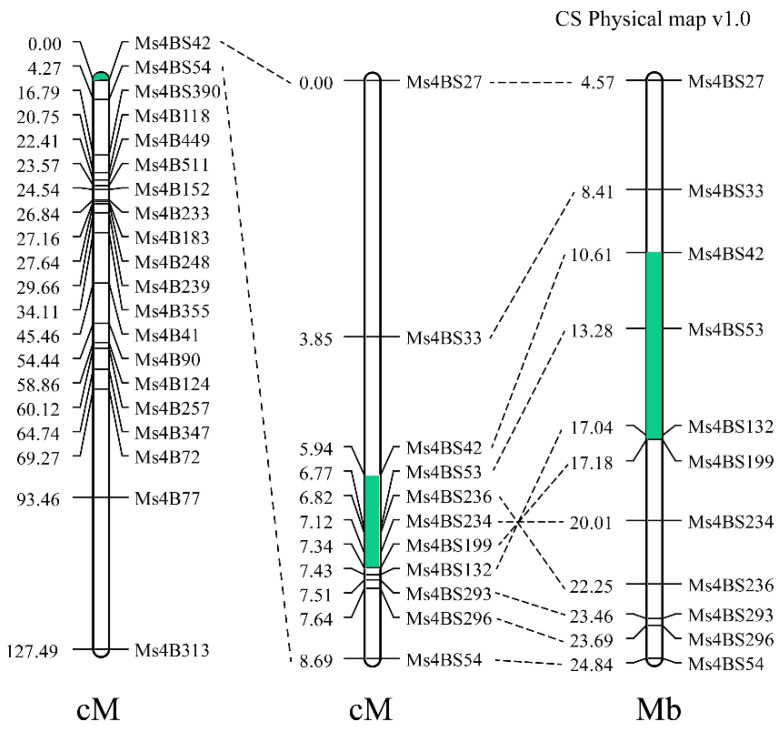
Linkage maps for locus *Ms1*. Green sectors portray the deleted chromosome 4BS segment, and the putative inverted segment is indicated.

To further map the gene-regulating male sterile of 15 Fan 03 line, we used 364 individuals from the F_2_ population and developed SSR markers in the predicted physical regions of chromosomes 4B and 5B. SSR markers *Ms4BS42*, *Ms4BS54*, *Ms4BS390*, and *Ms4B118* located at the terminal region of chromosome 4BS were significantly associated with sterility, whereas the markers for chromosome 5B were not (Figure 6). The genetic map positioned the sterility locus between SSR markers *Ms4BS42* and *Ms4BS54* in a genetic interval and physical distance of 4.27 cM and 14.23 Mb, respectively. Based on the Chinese Spring Reference Genome (IWGSCWGA v1.0), these markers were physically located on chromosomes 4BS at 10.61 and 24.84 Mb, respectively.

To narrow down the interval of the sterility locus, 1205 F_2_ individuals were screened by SSR markers *Ms4BS42* and *Ms4BS54*, and 184 recombinants were identified. New SSR markers developed from the target region allowed the locus to be fine-mapped between *Ms4BS42* and *Ms4BS199* at a genetic distance of 1.4 cM and a physical distance of 6.57 Mb. Based on IWGSCWGA v1.0, markers *Ms4BS42* and *Ms4BS199* were located on chromosomes 4BS at 10.61 and 17.18 Mb, respectively. Using the remaining polymorphic markers *Ms4BS132*, *Ms4BS199*, *Ms4BS234*, and *Ms4BS236*, the genetic distance of target region reduced. However, the physical distance based on IWGSCWGA v1.0 increased, indicating that the region between *Ms4BS132* and *Ms4BS236* of 15 Fan 03 line is significantly different from the Chinese Spring reference genome sequence.

### 2.3. Sterility in Line 15 Fan 03 Is Caused by a Chromosome Deletion

**Figure 7:**
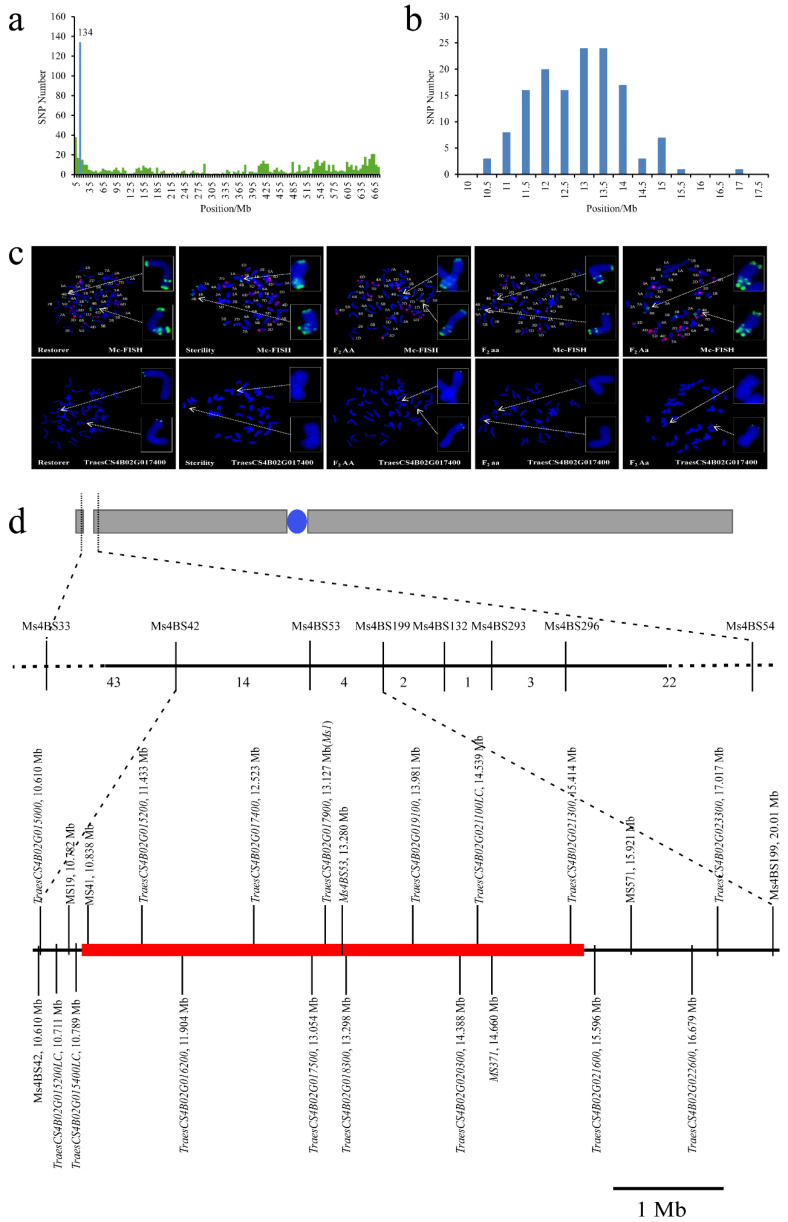
Chromosome deletion fragment identified by FISH in 15 Fan 03, Mian 07-374, and F_2_ plants. (**a**,**b**) Distribution of SNPs on chromosome 4B; (**c**) FISH analysis using (upper section) pSc119.2 (labeled green) and pAs1 (labeled red), and (lower section) *TraesCS4B01G017400* (labeled green) as probe. Arrows indicate chromosome 4BS terminal region identified in the same cells. Left to right: Mian 07-374, 15 Fan 03, homozygous dominant, heterozygous, and homozygous recessive F_2_ plants, respectively; (**d**) Markers, number of recombinants, predicted genes and their physical locations in the deleted fragment of wheat chromosome 4B.

Many newly developed chromosome-specific 4BS markers in the mapped interval were dominant makers. Therefore, Wheat660K SNP chip results of the fertile pool and the sterile pool of F_2_ generation were used, and 874 SNPs were detected in the 4B chromosome of the fertile plant pool but not in the sterile plant pool. Of these, 134 SNPs were clustered in the 10–15 Mb region of 4BS (Figure 7a). We speculated that a chromosome segment might be lost in this region.

Specific primers for 16 genes located close to the sterility gene were designed to verify the hypothesized deletion (Table 1 and Table S1). The first 10 sequentially distributed genes were not amplified in the sterile pool and sterile F_2_ individuals. According to the physical locations of the genes, it was predicted that a fragment of 5 Mb would be missing from the target region. The upstream break was between *TraesCS4B02G015400LC* and *TraesCS4B02G015200* (physical locations 10.79 and 11.43 Mb, respectively), and the downstream break was between *TraesCS4B02G021300* and *TraesCS4B02G021600* (physical locations 15.41 and 15.60 Mb, respectively).

Fluorescence in situ hybridization (FISH) was carried out to further verify the results. *TraceCS4B017400* was used as a probe to hybridize with the sterile line 15 Fan 03, fertile line Mian 07-374, and homozygous recessive sterile and dominant fertile F_2_ individuals (with homozygous and heterozygous individuals confirmed by SSR markers *Ms4BS42* and *Ms4BS54*). Mian 07-374 and homozygous fertile F_2_ individuals had two hybridization signals at the end of chromosome 4BS, heterozygous F_2_ individuals had one signal, and sterile plants from 15 Fan 03 and sterile F_2_ individuals had no signals (Figure 7c). We concluded that sterility was caused by deletion of a substantial fragment from chromosome 4BS.

In order to narrow down the location and size of the deleted fragment, a number of SSR markers were developed using IWGSCWGA v1.0 as reference. Primers MS13, MS19, MS571, MS572, and MS577 were amplified in all four pooled samples, whereas MS41, MS42, MS43, MS366, MS367 showed amplification only in Mian 07-374 and homozygous dominant fertile F_2_ individuals. Based on the physical location of the SSR markers on chromosome 4BS and the deletion interval, the upstream boundary of the deleted fragment was between *TraesCS4B025400LC* and the marker MS41, physically located at 10.789 and 10.838 Mb, respectively. The downstream boundary was predicted between the gene *TraesCS4B021300* and *TraesCS4B02G021600*, which are physically located at 15.409 and 15.596 Mb, respectively.

In previous studies [19,26], the dominant fertile allele at locus *MS1* was mapped to position 13.126 Mb on chromosome 4BS. This position falls within the deleted fragment found in sterile individuals of 15 Fan 03. Continuing the naming of mutant alleles at this locus causing sterility, the present allele is named *ms1s*.

### 2.4. Breeding Molecular Marker Development for ms1s

**Figure 8:**
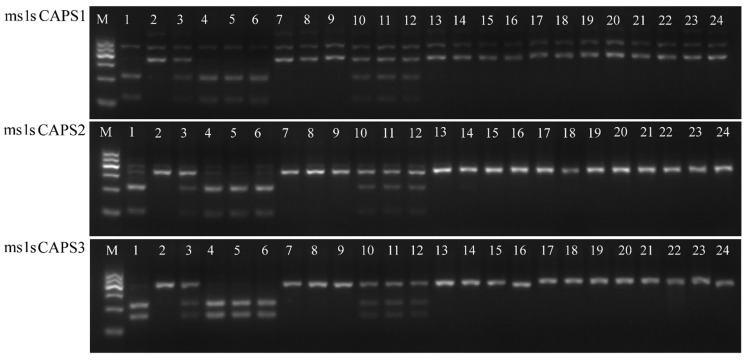
Images of CAPS markers in 1, sterile plant from 15 Fan 03; 2, Mian 07-374; 3, mixed pool of heterozygous F_2_ plants; 4–6, sterile F_2_ plants; 7–9, fertile F_2_ plants; 10–12, heterozygous F_2_ plants; 13–24; varieties Kenong 199, Zhoumai 27, Zhongyou 335, Zhengmai 9405, Jiyanmai 7, Zhongma 175, Xumai 0054, Zhongmai 8, Liangxing 99, Zhongmai 998, Goumai 301, Lankao 182.

The Wheat660K SNP chip data identified SNP *AX-111474959* (physical position 10.615 Mb) as the closest SNP to the sterility gene. Three CAPS markers (*ms1sCAPS1*, *2*, and *3*) were developed from the flanking region of this SNP based on IWGSCWGA v1.0. Restriction enzyme Bgl II (NEB, CITY, USA States) cleaved PCR products only from sterile individuals from 15 Fan 03 and sterile F_2_ individuals (*ms/ms*) into two fragments. The ms1sCAPS1 cleaved an amplified 361 bp region into 232 bp and 129 bp fragments; ms1sCAPS2 cleaved an amplified 347 bp product into 232 bp and 115 bp fragments; and ms1sCAPS3 cut a 395 bp fragment into 232 bp and 163 bp fragments (Figure 8).

Pooled DNA from random F_2_ individuals and heterozygous F_2_ individuals produced three fragments after the restriction enzyme Bgl II treatment, of which ms1sCAPS1 produced 361 bp, 232 bp, and 129 bp; ms1sCAPS2 produced 347 bp, 232 bp, and 115 bp; and ms1sCAPS3 produced 395 bp, 232 bp, and 163 bp fragments. These results confirmed the functionality of the three CAPS markers for the selection of male sterility allele *ms1s*.

## 3. Discussion

Application of nuclear male sterility is one way to study heterosis in wheat. Although there are many reports on nuclear male sterility in wheat, only 5 loci *Ms1-Ms5* have been identified and physically mapped. Sterility-causing alleles of *Ms1* and *Ms5* are recessive, and those of *Ms2*, *Ms3*, and *Ms4* are dominant. Three of them have been isolated and cloned. The dominant *Ms2* sterility allele in *Taigu* contains an originally unexpressed “orphan” gene in its promoter region, which is activated and specifically expressed in anthers causing male sterility [13]. Tucker et al. (2017) and Wang et al. (2017) cloned the wildtype allele of *Ms1* and found that it encodes a lipid transport protein expressed only in microspores [19,26]. Pallotta et al. (2019) demonstrated that *Ms5* encodes a lipid transfer protein anchored by glycosylphosphatidylinositol, which is required for normal development of the pollen outer wall [12].

Here, we studied the wheat nuclear male-sterile line 15 Fan 03 and compared it with Mian 07-374 and generated an F_2_ population. The sterility gene was mapped between the SSR markers *Ms4BS42* and *Ms4BS199* physically positioned at 10.61 and 17.18 Mb, respectively. Sterility was caused by deletion of a chromosome 4BS fragment, detectable by chromosomal FISH. Based on IWGSCWGA v1.0, the deleted region included *TraesCS4B01G017900* identified as the *Ms1* allele positioned at 13.126 Mb. Among reported *Ms1* mutants, *ms1a* (Pugsley’s mutant) and *ms1c* (Cornerstone) mutants are terminal deletions of chromosome 4BS [18,27]; *ms1b* (Probus mutant) and *ms1g* (LZ, [25]) are interstitial deletions [17,19]; and *ms1d* (FS2), *ms1e* (FS3), and *ms1f* (FS24) are nucleotide variants [10]. Nucleotide variant alleles in a series of EMS-induced sterile mutants in variety Ningchun 4 were named *ms1h*, *ms1i*, *ms1j*, *ms1k*, *ms1l*, *ms1m*, *ms1n, ms1o, ms1p,* and *ms1q* [19]. At the same time, a nucleotide variant allele in a Qual 2000 TILLING population was named *ms1h*. This allele has been re-designated as *ms1r* (RA McIntosh, personal communication).

The flowering phenotype of sterile plants in 15 Fan 03 differs from that in the fertile plants of Mian 07-374, as the glume remains open for a longer time at a wider angle, and at the tri-nuclear stage, the pollen grains degrade and the pollen activity is lost. However, genetically, the sterile line 15 Fan 03 was similar to *ms1b* and *ms1g* mutants in possessing interstitial deletions of 4BS chromosome segments bearing the *Ms1* locus.

Wheat nuclear male-sterile lines have many advantages. For example, they are easily restored by most common wheat varieties, with significant heterosis, and without the adverse effect of exogenous cytoplasm. Furthermore, it is easy to select excellent hybrid combinations. The main problem with this type of male-sterile line is that it is difficult to obtain a stable male-sterile maintainer line; that is, it is difficult to produce a large number of homozygous male-sterile seeds. However, using the blue-aleurone nuclear male-sterile maintainer system (BM system), we can easily obtain wheat male-sterile lines (white grains, such as 15 Fan 03) and maintainer lines (light blue grains). We obtained 11 combinations of BM systems with modern main varieties. Moreover, white and blue seeds can be separated at the seed stage, so it is easier to obtain pure male-sterile lines for the production of hybrid wheat [23,24]. This solves the difficulties in obtaining stable male sterile maintainer lines and producing a large number of homozygous male sterile seeds. Moreover, the easy restoration of these lines by common wheat varieties has opened up a new direction for the application of and research into wheat nuclear male sterility.

Because the genome of common wheat is large, complex, and heterogeneous, many chromosomal variations are preserved in the species. These chromosomal abnormalities may also alter the plant phenotype. For example, the absence of the end of chromosome 5AL causes reduced plant height [28]. In this study, a deleted chromosomal fragment was found in sterile plants, and the positions of some markers were in opposite orientations in genetic and physical maps (Figure 6), indicating that chromosomal inversion might have occurred in the parental material. The upstream position of the deletion was between *TraesCS4B02G015400LC* and marker *MS41*, whereas the downstream position was between *TraesCS4B021300* and *TraesCS4B02G021600.* Using this information and the wheat660K SNP chip data, three CAPS markers were developed and validated on the F_2_ pooled DNA and heterozygous F_2_ individuals, as well as 12 widely grown modern wheat varieties. These CAPS markers can be used to genotype individuals in segregating populations at an early stage of growth. Since an SNP marker is usually developed from a sequence polymorphism between different cultivars, it is background genotype specific. We are currently exploring other types of molecular markers that might be more suitable for marker-assisted selection of *ms1s*.

## 4. Materials and Methods

### 4.1. Plant Material

An F_2_ population of 1205 individuals derived from a cross between 15 Fan 03 (white grain, a nuclear male-sterile line, derived from the mutant in the offspring of a cross of wheat varieties 72,180 and Xiaoyan 6, which introduced a 4E chromosome from *Agropyron elongatum* containing a dominant blue endosperm gene and a fertility restorer gene) [24] and Mian 07-374 was used for genetic analysis, linkage mapping, and gene localization. At least three spikes from each plant at heading were bagged, and the seed-setting rate was calculated as follows: Seed setting rate=(seeds from the two florets of the spikelet base)⁄(no. of spiklets)×2. The seed setting rates lower than 10% represent sterile individuals; the seed setting rates higher than 10% correspond to fertile ones.

### 4.2. Morphological Observations

15 Fan 03, Mian 07-374, and the F_2_ population were planted in 1.5 m rows, with plant-to-plant and row-to-row spacings of 10 cm and 25 cm, respectively, at the experimental farm of Institute of Genetics and Developmental Biology in 2017. Anthers at the mature tri-nuclear stage were sampled and stained with 1% I_2_-KI solution for 10 min to determine the pollen fertility.

Anthers for histological studies were excised at different developmental stages, fixed in FAA solution for 24 h, sliced into 4 mm sections, stained with 1% aniline saffron and 0.5% aniline fast green, and observed under bright field using a Nikon Eclipse Ci-S microscope. For scanning electron microscopy (SEM), anthers at the tri-nuclear pollen stage were dried using a CO_2_ dryer (Hitachi Critical Point Dryer HCP-2) for 30 mins, and the golden surface of the dried anthers was studied using a Hitachi S-3000N electron microscope.

### 4.3. Wheat660K SNP Chip Analysis

Genomic DNA from 15 Fan 03 (male-sterile line), Mian 07-374 (fertile line), and the F_2_ population was extracted using a plant genomic DNA extraction kit (DP305, Tiangen Biochemical Technology Co., Ltd., Beijing, China). Four DNA pools, separately derived from 10 sterile plants of 15 Fan 03, the fertile line, F_2_ individuals with more than 99% seed set, and F_2_ individuals with 0% seed set, were pooled and scanned by the Wheat660 SNP chip for genome-wide SNP analysis [29]. Sequences flanking SNPs were blasted against IWGSCWGA v1.0 to predict physical locations on the Chinese Spring Reference Genome. The SNPs identified in the four DNA pools were statistically analyzed using Microsoft Excel.

### 4.4. SSR Marker Development and Genetic Map Construction

Genomic DNA of the F_2_ individuals was extracted using the CTAB method [30]. SSRLocator software [31] was used to develop SSR markers based on IWGSCWGA v1.0. The parameters of primers were set at 18–23 bp, annealing temperature (Tm) 55–63 °C, GC content 50–60%, and amplified DNA sequence length 100–280 bp (Table 2). All the markers developed within the mapping interval were used for genotyping. The linkage map was constructed using JoinMap 4.0 software [32] and MapChart 2.30 software [33].

### 4.5. Fluorescence In Situ Hybridization

Root tips were prepared for fluorescent in situ hybridization (FISH) as described by Han et al. [34]. The genomic DNA of *TraesCS4B01G017400* was amplified using the following primer pair: forward, 5′-GGGCTGGGAAAGAGCA-3′ and reverse, 5′-CCCCAAGATACGTGTTCC-3′. It was labeled with Alexa Fluor-488-dUTP (green) as needed. For Mc-FISH (Tang et al. 2014), pAs1 and pSc119.2 were labeled with Alexa Fluor-488-dUTP (green) and Alexa Fluor-594–5-dUTP (red), respectively. The FISH images were acquired using the epifluorescence ZEISS AXIO Imager 2.0.

## Figures and Tables

**Table 1:** Primers specific to the 16 genes in the male-sterile gene location interval.

Gene ID	Forward Primer	Reverse Primer	Position (Mb)
TraesCS4B01G015000	GCCTATCTTTGAACCGGCGA	AATTAGGCGAGCCCAAGAGC	10.610
TraesCS4B01G015200LC	CCCGGGCTTATGAAGGCTTG	ATGGAAGGAGGAGGCATTGA	10.712
TraesCS4B01G015400LC	GTATGCGACGACTCCCGAAG	AGTACGCCGACTTCCCCTAT	10.789
TraesCS4B01G015200	CGTCGGACGATACGGACACA	TGTCACCCTCCATCCTACGTAC	11.433
TraesCS4B01G016200	CGCCGGTAGGTAGTAATCCG	TCCTGAGCCTGACCATCTCG	11.904
TraesCS4B01G017400	CCCTGTGCGGAATCTTCCAA	CCAGAGTCACAAACCGCTCA	12.526
TraesCS4B01G017500	ACATTCACTTGACAGCGACCT	CCACGGTACTCGCATGCTC	13.053
TraesCS4B01G017900 (*Ms1*)	ACGCAGAAACGAACCTCGAA	GCTAGAAGTGCTCCGGTTTG	13.126
TraesCS4B01G018300	TACTTGTCGGCCAAACGGA	CGTCCGTCAAGAACACACCA	13.297
TraesCS4B01G019100	GTGGAGCAAGTGGACTCACC	CCAGGCAAGTCGTCGATTCA	13.977
TraesCS4B01G020300	GAAAACCCTAGACGCTCCCC	GCACCAACAAAACCGTCAGA	14.389
TraesCS4B01G021100LC	ACTTTTGCGCCAGGTATCCA	GCCTCTCGCGAAAGAAATGC	14.539
TraesCS4B01G021300	GGCATGTGCCTAGTGGGAAT	CAGCAGCACAAGGTTGGTTC	15.409
TraesCS4B01G021600	GAGGTCATTTACCAGTGCCAGT	TTCCACCGGTACTCTTCAGC	15.596
TraesCS4B01G022600	GGGCTACCGGCAACGTAATA	AAGCGCACCCTCACAAGAAA	16.680
TraesCS4B01G023300	CGCTGGTTGGAGTTTACCCT	TGTAAGGTCTTAGCGCCCAC	17.017

**Table 2:** CAPS primers developed in this study.

Primer	Sequence (5′-3′)	Product Size (bp)
ms1sCAPS1F	AAGAGCCCATATGCACCTTG	361
ms1sCAPS1R	GGTACCAGGGGGTTGAAGAT	
ms1sCAPS2F	AAGAGCCCATATGCACCTTG	347
ms1sCAPS2R	GAAGATAACTGGGCGTGGAA	
ms1sCAPS3F	AAGAGCCCATATGCACCTTG	395
ms1sCAPS3R	TGCGAAAGTGTTGTGCTACC	

## Data Availability

The datasets used and/or analyzed during the current study are available from the corresponding author on reasonable request.

## Supplementary Materials

Supplementary Materials: The following are available online at https://www.mdpi.com/article/10.3390/ijms22168541/s1, Table S1: Information of candidate genes in co-segregation region of ms1s.
